# Impact-Oriented Dialogue for Culturally Safe Adolescent Sexual and Reproductive Health in Bauchi State, Nigeria: Protocol for a Codesigned Pragmatic Cluster Randomized Controlled Trial

**DOI:** 10.2196/36060

**Published:** 2022-03-15

**Authors:** Anne Cockcroft, Khalid Omer, Yagana Gidado, Rilwanu Mohammed, Loubna Belaid, Umaira Ansari, Claudia Mitchell, Neil Andersson

**Affiliations:** 1 Community Information for Empowerment and Transparency-Participatory Research at McGill Department of Family Medicine McGill University Montreal, QC Canada; 2 Centro de Investigacion de Enfermedades Tropicales Universidad Autonoma de Guerrero Acapulco Mexico; 3 Federation of Muslim Women's Associations of Nigeria Bauchi Nigeria; 4 Bauchi State Primary Health Care Development Agency Bauchi Nigeria; 5 Department of Integrated Studies in Education McGill University Montreal, QC Canada

**Keywords:** adolescents, sexual and reproductive health, participatory research, mixed methods research, dialogic intervention, co-design, cultural safety, Nigeria

## Abstract

**Background:**

Adolescents (10-19 years) are a big segment of the Nigerian population, and they face serious risks to their health and well-being. Maternal mortality is very high in Nigeria, and rates of pregnancy and maternal deaths are high among female adolescents. Rates of HIV infection are rising among adolescents, gender violence and sexual abuse are common, and knowledge about sexual and reproductive health risks is low. Adolescent sexual and reproductive health (ASRH) indicators are worse in the north of the country.

**Objective:**

In Bauchi State, northern Nigeria, the project will document the nature and extent of ASRH outcomes and risks, discuss the findings and codesign solutions with local stakeholders, and measure the short-term impact of the discussions and proposed solutions.

**Methods:**

The participatory research project is a sequential mixed-methods codesign of a pragmatic cluster randomized controlled trial. Focus groups of local stakeholders (female and male adolescents, parents, traditional and religious leaders, service providers, and planners) will identify local priority ASRH concerns. The same stakeholder groups will map their knowledge of factors causing these concerns using the fuzzy cognitive mapping (FCM) technique. Findings from the maps and a scoping review will inform the contextualization of survey instruments to collect information about ASRH from female and male adolescents and parents in households and from local service providers. The survey will take place in 60 Bauchi communities. Adolescents will cocreate materials to share the findings from the maps and survey. In 30 communities, randomly allocated, the project will engage adolescents and other stakeholders in households, communities, and services to discuss the evidence and to design and implement culturally acceptable actions to improve ASRH. A follow-up survey in communities with and without the intervention will measure the short-term impact of these discussions and actions. We will also evaluate the intervention process and use narrative techniques to assess its impact qualitatively.

**Results:**

Focus groups to explore ASRH concerns of stakeholders began in October 2021. Baseline data collection in the household survey is expected to take place in mid-2022. The study was approved by the Bauchi State Health Research Ethics Committee, approval number NREC/03/11/19B/2021/03 (March 1, 2021), and by the Faculty of Medicine and Health Sciences Institutional Review Board McGill University (September 13, 2021).

**Conclusions:**

Evidence about factors related to ASRH outcomes in Nigeria and implementation and testing of a dialogic intervention to improve these outcomes will fill a gap in the literature. The project will document and test the effectiveness of a participatory approach to ASRH intervention research.

**Trial Registration:**

ISRCTN Registry ISRCTN18295275; https://www.isrctn.com/ISRCTN18295275

**International Registered Report Identifier (IRRID):**

DERR1-10.2196/36060

## Introduction

### Background

Pregnancy and childbirth complications are the leading cause of death in girls aged 15 to 19 years in low- and middle-income countries [1]. Adolescent sexual and reproductive health (ASRH) risks in sub-Saharan Africa are extremely high, with important intersectional inequalities related to gender, education, economic status, and urban/rural residence [2].

One-fifth of Nigeria’s population are adolescents aged between 10 and 19 years, and the government recognizes the need to protect their sexual and reproductive health [3]. Adolescent girls are at higher risk. By the age of 18 years, 43% of Nigerian women are married, 56% have had sexual debut, and 29% have given birth [4]. In 2018, 19% of girls aged 15 to 19 years had begun childbearing, and 40% of deaths in this age group were related to pregnancy and childbirth [4]. Poor, uneducated women in rural areas are more likely to marry and to give birth early [4-6]. Pregnant teenagers may risk unsafe abortions [7,8]. Nigerian adolescents also face risks of HIV and gender violence [9-11]. Nearly all youth aged 15 to 19 years have heard of AIDS, but few have functional knowledge about prevention [4]. Nationally, 18% of women aged 15 to 19 years reported physical violence in the last year and 5% during their last pregnancy [4].

To protect adolescents, the Government of Nigeria introduced the Family Life and HIV Education program in schools in 2003. The program only reaches a small proportion of in-school adolescents, and teachers are uncomfortable dealing with the topics [12,13]. Programs reach even fewer out-of-school youth [14].

Small studies describe the knowledge and experiences of Nigerian adolescents of sexual and reproductive health risks [15-21]. They rarely use available reproductive health services [22,23]. A recent scoping review of 1302 articles on ASRH in sub-Saharan Africa found studies most frequently focused on HIV, sexual behavior, and access to services. Few included younger adolescents (10-14 years), 53% used quantitative methods only, 44% were cross-sectional studies, and only 13% used mixed methods [24]. Many ASRH programs are ineffective [25], and there is a need to measure the impact of interventions and to engage adolescents in designing and implementing programs [26]. We are not aware of published studies that involved Nigerian adolescents in codesigning interventions to address their own sexual and reproductive health needs.

This project aims to fill this evidence gap. In a participatory approach, we will collect qualitative and quantitative evidence about ASRH and share it with adolescents and other stakeholders, who will codesign interventions to improve ASRH. The work builds on an existing strong collaboration between the research team and the state government. This collaboration completed two projects in Bauchi State under the Innovating for Maternal and Child Health in Africa initiative [27]: a trial of universal home visits to improve maternal and early childhood health [28], and a study of causes and prevention of short birth interval [29]. These projects included married adolescent girls aged 14 to 19 years.

### Conceptual Framework

The project considers influences on the sexual and reproductive health of adolescent girls and boys at individual, family, and broader structural levels [30]. At the individual and family level, the CASCADA (Conscious knowledge, Attitude, Subjective norms, intention to Change, Agency to change, Discussion of issues, and Action to change) results chain [31,32] expands the knowledge-attitudes-practice model [33], criticized for its lack of detail between attitudes and practice [34]. A modified theory of planned behavior [35], CASCADA is an acronym for a partial order of intermediate outcomes between knowledge and action. We have used CASCADA in resource-poor settings to support analysis of studies of health behaviors [36,37], in the design of interventions [38], and as a framework for analysis of intervention effects [39,40].

Many risks for ASRH are structural; potential solutions need to address factors that constrain individual choice, including patriarchy and unhelpful gender norms [30,41-43]. We developed a concept of *choice disability* among marginalized young women at high risk of gender violence and HIV in Southern Africa [44], showed its association with HIV risk [45], and implemented interventions combining individual empowerment, more responsive services, and the creation of an enabling environment to support individual choices [46,47]. Choice disability likely also applies to at-risk adolescents in Bauchi, although the cultural context is different.

The concept of *cultural safety* [48] requires that the people concerned—in this case, adolescents and other stakeholders in Bauchi—decide whether a service or intervention respects their cultural identity and values [49]. Beyond being a moral and ethical issue, cultural safety makes an intervention more likely to succeed.

*Participatory research* partners with and values the knowledge of stakeholders [50]. The project in Bauchi will prioritize and systematize the concerns and knowledge of local stakeholders, especially adolescents, about ASRH risks and engage them to codesign and implement solutions that work within their cultural context. Our recent work on *kunika* (short birth interval) in Bauchi demonstrates how an approach of integrated knowledge translation and exchange can result in locally identified and culturally attuned interventions and communication materials for a sensitive issue (U Ansari, unpublished data, January 2022) [29,51].

### Research Goal and Objectives

#### Goal

Codesign culturally appropriate interventions to improve ASRH and pilot their implementation in Bauchi State, Nigeria.

#### Objectives

1. Explore priority stakeholder concerns about ASRH in Bauchi, collate the knowledge of female and male adolescents and other stakeholders about causes and protective factors for these concerns, and compare their knowledge with documented associations in the literature.
2. Quantify ASRH-related knowledge, attitudes, experiences, and behaviors of female and male adolescents, parents, and service providers, using data collection instruments informed by the collated local knowledge and literature review.
3. Engage adolescents, parents, service providers, and decision-makers in dialogue about the evidence on ASRH outcomes and causes, to identify and implement locally appropriate interventions at different levels to improve ASRH.
4. Evaluate the intervention process and measure impact on ASRH knowledge, attitudes, experiences, and behaviors of female and male adolescents and other stakeholders quantitatively and qualitatively.

### Study Design

The participatory research project is a sequential mixed-methods codesign of a pragmatic cluster randomized controlled trial. It will begin with qualitative data collection and a scoping review, supporting codesign of the dialogic intervention to share local findings about ASRH risks and codesign solutions with adolescents and other stakeholders. The impact evaluation of the intervention will use both quantitative and qualitative methods.

## Methods

### Setting

The study will take place in communities in the Toro Local Government Area of Bauchi State in the North East of Nigeria. Ninety-five percent of the state population are Muslim. Health and education indicators are worse than elsewhere in Nigeria. Only 26% of women and 48% of men aged 15 to 49 years are literate in Bauchi State, compared with 53% and 72% nationally [4]. Women in Bauchi start childbearing particularly young; 41% of girls aged 15 to 19 years have given birth or are pregnant [4]. In a recent program of home visits in Bauchi State [28], 56% of 7282 women aged 14 to 49 years were pregnant during one year, and 38% of those aged 14 to 19 years. Gender violence is common in Bauchi. In one study, 23% of women aged 14 to 19 years experienced domestic violence in the last year and 15% during their last pregnancy [52].

### Qualitative Exploration of ASRH Concerns and Scoping Review

#### Focus Group Discussions

Local researchers will facilitate focus group discussions with female and male adolescents and other stakeholders about priority concerns for ASRH. In each participating community, they will conduct 10 groups: 5 adolescent groups, 2 groups of parents of adolescents (male and female), 2 groups of traditional and religious leaders (male and female), and 1 group of service providers (mixed male and female). The adolescent groups will comprise 3 female groups (10-14 years, 15-19 years unmarried, and 15-19 years married) and 2 male groups (10-14 years and 15-19 years). All groups will include adolescents in and out of school. Female team members will facilitate female groups, and male team members will facilitate male groups. Young facilitators (less than 25 years) will facilitate the groups of adolescents. The 6 participating communities will be 2 urban, 2 rural, and 2 rural-remote, spread across 6 wards in Toro Local Government Area. The research team will also conduct 2 groups with male and female ward-level leaders in each of the 6 wards, 1 group with government officers in the local government area, and 3 groups at the state level, with health planners, traditional and religious leaders, and nongovernment organizations (NGOs). In total, there will be 76 focus groups and 380 to 456 participants (5-6 per group).

#### Analysis

An inductive thematic analysis will identify priority ASRH concerns [53], using the approach of Braun and Clarke [54] and applying criteria for trustworthiness proposed by Guba [55,56]. Without pre-empting the findings, we expect that the priority ASRH concerns will likely include adverse effects of early pregnancy, sexually transmitted infections, gender violence, sexual abuse, and poor access to responsive services.

#### Fuzzy Cognitive Mapping

Fuzzy cognitive mapping (FCM) is the graphic representation of knowledge about causality in a system [57,58]. FCM depicts factors that stakeholders consider to be causes of an outcome, in this case, adverse ASRH outcomes. The stakeholders rate the strength of associations between these factors and the outcome. We will compare maps between different stakeholder groups using the mathematical technique of transitive closure [59,60].

Local fieldworkers will facilitate stakeholder groups to create maps of the factors they believe cause the priority ASRH concerns identified in the focus groups. The stakeholders will estimate the strength of each association in the map. They will use a scale of 1 to 5, with 5 representing the strongest association. The FCM groups will be the same as the focus groups.

#### Analysis

We will digitize the maps using YEd software [61]. A thematic analysis [53,54] of factors in the maps will create broader themes. Transitive closure analysis [59,60] will identify the most influential factors. The analysis will give the knowledge of adolescent girls and boys at least equal weight to that of other stakeholders. We will compare maps of different stakeholder groups and maps from stakeholder groups with the map from the scoping review [62]. Causes of adverse ASRH outcomes identified on the maps will inform the contextualization of the survey instruments. Based on our experience of FCM of causes of short birth interval in Bauchi, we expect they will include causes at individual, family, community, and service levels [29].

### Scoping Review of the Literature

Systematic reviews have examined the effectiveness of interventions to improve ASRH, including in low- and middle-income countries [25,26,63]. A 2020 review of studies and demographic and health survey data from sub-Saharan Africa confirmed high ASRH risks, with variation between countries [2]. There remains a need to collate quantitative and qualitative evidence of associations with ASRH in low- and middle-income countries. Supported by a specialist librarian, we will conduct a scoping review of quantitative and qualitative studies of factors associated with ASRH outcomes in low- and middle-income countries. In addition to a standard reporting of the review, we will create a fuzzy cognitive map (see as follows) of factors related to ASRH outcomes, marking the strength of associations as odds ratios or regression coefficients, and compare the literature map with stakeholder-created maps [62,64,65].

### Cluster Randomized Controlled Trial of Dialogic Intervention to Promote ASRH

#### Overview

The trial will begin with a baseline household survey in all study communities. Half the communities will participate in evidence-based dialogues to plan and implement local solutions to ASRH concerns. Normal health and other services will continue in all communities. A follow-up household survey in all communities will document the quantitative impact of the intervention on priority ASRH outcomes, and narratives of change will explore perceived experiences of the intervention.

#### Participants

The household sample for measurement of impact in intervention and control communities will comprise all adolescents and their parents in 100 households in each community. Eligible households will have at least one adolescent girl. We will adjust survey timing to ensure we reach in-school as well as out-of-school adolescents. No adolescent or parent who agrees to participate in the survey will be excluded. For the intervention, all adolescents, adults, service providers, and traditional and religious community leaders in the intervention communities will be eligible to participate in activities developed in each community after discussing the local evidence in dialogue groups.

#### The Intervention: SEPA

We have developed the socializing evidence for participatory action (SEPA) approach over 25 years [32] to support participatory action on health concerns [38,66-69]. The SEPA approach includes deliberative dialogue where small groups of stakeholders discuss local evidence and decide on actions to tackle a common concern [70]. SEPA is itself an intervention, and we can measure its impact. Stakeholders decide what actions to take; these vary from place to place, but the protocol of sharing and discussing evidence can be standardized and randomized [71].

#### Evidence Materials

We will prepare summarized outputs from the transitive closure analysis of the cognitive maps [29]. With support from the research team, groups of approximately 15 adolescent girls and boys respectively, identified through a local NGO, the Federation of Muslim Women’s Associations in Nigeria (FOMWAN), will produce materials to share evidence from the maps and baseline survey, including short video docudramas in the style of popular local soap operas. We recently codesigned with community groups video docudramas about short birth intervals in Bauchi (U Ansari, unpublished data, January 2022). We will work with the female and male adolescents and a local production company to prepare the video docudramas and other communication materials in about four months at the end of year two.

#### Implementation of the Intervention

SEPA will take place over 18 months, in years three and four. Local researchers and adolescents will share the findings about ASRH, including sharing the video docudramas, with groups in the 30 SEPA communities: adolescent girls, adolescent boys, male and female adults, community and religious leaders, and relevant service providers. The groups will plan and implement local actions to improve ASRH in their communities, engaging other adolescents and other stakeholders in these community actions. The research team will provide logistic and administrative support for the community groups and their actions. Research team members will visit the communities monthly to liaise with community leaders, document progress, and help to resolve challenges. Follow up will be virtual if necessary; we have successfully used cellular teleconferencing in Bauchi communities [72]. Adolescent girls and boys will meet separately; we will explore ways to share ideas between female and male groups, for example, creating visual media such as cellphilms [73]. In combined dialogue groups, there is a risk that powerful stakeholders could silence the voice of adolescents, especially females. Adolescents and other stakeholders will initially meet separately; our trained facilitators will only convene combined groups if the adolescents are confident, and we will give them a protected space to speak in any combined groups. We expect that actions proposed by the SEPA groups will engage larger numbers of adolescents and other stakeholders. To support changes in service provision and community norms, we will include service providers and powerful stakeholders; some may change their views through their engagement. The research team will broker dialogues between adolescents, adults, and service providers in ways that recognize the steep power gradients involved and take steps to ensure that the voices of adolescents, especially female adolescents, are heard and respected. About 500 adolescents will participate in community SEPA groups, and we expect perhaps 3000 to participate in community activities led by the SEPA groups.

#### Process Evaluation of the SEPA Intervention

The process evaluation will consider implementation, mechanisms, and context [74]. Towards the end of the implementation period, adolescents and other stakeholders implementing SEPA in each community will review their achievements and document the challenges they faced. Using reports from these meetings and documentation of the SEPA activities over the period, a researcher not involved in implementation will “score” the level of implementation in each community, based on level of participation in groups, planned activities, and activities that were implemented. This will include adolescent-led activities as well as the support or resistance they received from other stakeholders in the community and services. This implementation score will be a factor in the quantitative analysis of the impact of the intervention.

#### Outcomes

The initial focus groups and FCM with adolescents and other stakeholders will inform the specific priority ASRH outcomes to be addressed and measured. The outcomes are likely to include sexually transmitted diseases, experience and perpetration of physical and sexual violence, emotional distress, and use and experience of health services. Intermediate outcomes will cover steps in the CASCADA sequence. Questionnaires administered to adolescents and parents in the baseline household survey and the follow-up household survey 18 to 24 months later will collect data to measure the outcomes.

#### Timing

**Figure 1:**
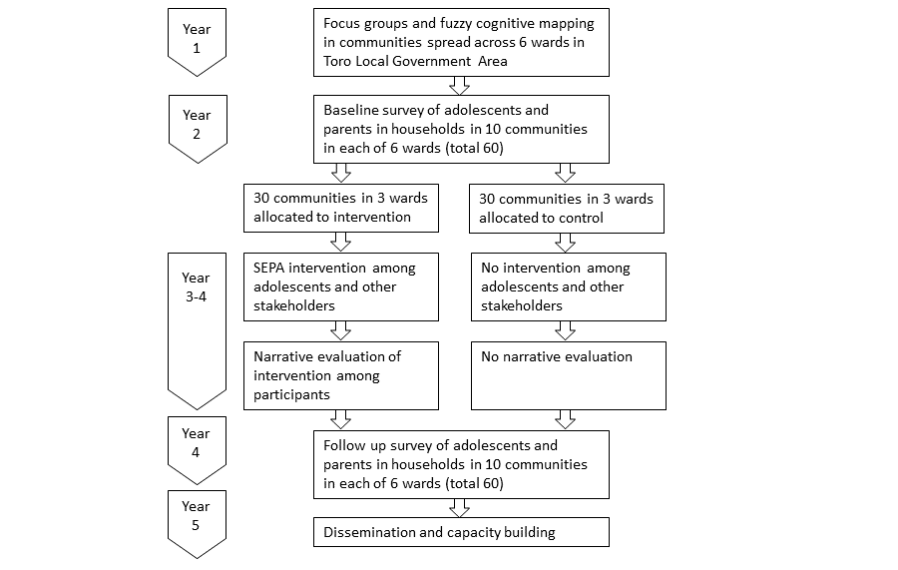
Timeline of the project. SEPA: socializing evidence for participatory action.

Figure 1 shows the timeline for the project, including the initial qualitative phase and the trial of the intervention.

#### Sample Size

The sample for the baseline and follow-up household survey will include 6000 adolescent girls and 4000 adolescent boys, plus 4000 mothers and 2500 fathers. The clinical trials simulator of Taylor and Bosch [75] estimates study power for the indicative outcome of the experience of violence by adolescent girls. With an expected prevalence of about 20% at baseline, and a sample of 3000 girls in 30 SEPA communities and 3000 in 30 non-SEPA communities, a 15% reduction of gender violence (to 17%) would be detectable with 80% power at the 5% level (κ=0.05). We anticipate a household response rate of around 95%. In a previous Bauchi household survey, the response rate was 96% (3.4% were not available, and 0.4% declined) [76].

#### Allocation of the Intervention

We will stratify the wards by size and proportion of urban communities, and an epidemiologist not involved in the fieldwork will randomly allocate (using a computer-generated random sequence) 3 of the 6 wards to receive the SEPA intervention. All the 30 study communities in the 3 SEPA wards will participate in SEPA. We do not expect any community to decline to participate; they all participated in and strongly valued our recent home visits program [40,77].

#### Blinding

It is not possible to blind participating communities to the SEPA intervention. The dialogue groups and resulting community activities will be apparent and are intended to be. The interviewers for the household surveys will not be involved in supporting intervention activities. They may become aware of the intervention status of some communities when undertaking the follow-up survey; they will use a standard questionnaire in all communities, and there is no reason to believe they will conduct interviews differently in intervention and control communities.

#### Quantitative Data Collection and Analysis

The baseline survey will take place in year two of the project in 60 communities in the 6 wards of the Toro Local Government Area. In each community, a team of interviewers will cover a cluster of 100 households, recording questionnaire responses on android handsets using ODK software (Get ODK) [78]. They will upload records to a Cloud server via the cellular network, and we will download data sets for analysis. The analysis will use CIETmap open-source software (version 2.2.21; CIET group) [79], which interfaces with the R programming language (R Core Team). The household survey of adolescents and parents will provide data on the frequency of the specified ASRH outcomes and individual and family factors potentially related to these. Institutional reviews of health facilities serving the communities will provide data about potential supply-side determinants. Key informant interviews with community leaders and opinion-makers and community profiles will record factors at the community level potentially relevant to ASRH outcomes.

The follow-up survey will cover the same 60 communities as the baseline survey, but not necessarily the same households.

##### Data Collection Instruments

1. ASRH questionnaires for adolescents and adults will draw on existing validated instruments [80-82], contextualized by findings of the focus groups and FCM. They will include intermediate outcomes of knowledge, attitudes, and experience amenable to change stimulated by the intervention in a short time scale.
2. A guide for institutional reviews of health facilities will gather information on available ASRH services in one clinic per community
3. A questionnaire for key informants and a community profile proforma will enquire about factors relevant to ASRH at the community level. Key informants will include traditional and religious leaders, headteachers, health facility heads, and social workers.

##### Quantitative Data Analysis

Bivariate and then multivariate analysis of responses to the baseline survey will examine associations between ASRH outcomes and potential determinants at individual, family, and community/services level, using the Mantel-Haenszel procedure [83], with the Lamothe cluster adjustment [84]. The analysis will include gender as a key variable in the examination of ASRH outcomes and associations with these outcomes.

To measure the impact of the intervention, we will compare the pre-specified ASRH outcomes at follow-up between adolescents in SEPA and non-SEPA wards and compare the change from baseline to follow-up between SEPA and non-SEPA wards. We will use generalized estimating equations to account for clustering (at ward and community levels), differences at baseline, known potential confounders, and any other community-level changes in the period [85]. A supplementary analysis will look at changes in outcomes in relation to the SEPA implementation ‘score’ (see Process Evaluation of the SEPA Intervention) in individual SEPA communities.

#### Qualitative Evaluation of Impact

Drawing on the most significant change technique [86], we will collect stories of life changes that participants attribute to the SEPA intervention. These stories can reveal both expected and unexpected effects of the intervention. This narrative approach complements quantitative evaluations, shedding light on possible mechanisms of effect. Sampling is usually purposive and should aim to include a range of experiences and views [87,88].

In intervention communities, local researchers will identify a purposive sample of 60 storytellers, including at least 25 adolescent girls and 15 adolescent boys. The sample will include people likely to have had a range of different experiences depending on their age, gender, urban or rural residence, role in the community, and extent of involvement in SEPA. Fieldworkers will collect stories by taking notes as the storytellers speak, reading back the stories to them to check for accuracy.

A hybrid thematic analysis [53,54] of the narratives will include a deductive analysis using the CASCADA results chain as a framework, supplemented by an inductive analysis of other themes emerging in the narratives.

#### Auditing Trial Conduct

A project steering committee led by the Bauchi State Primary Health Care Development Agency and including relevant government bodies and the research team will meet twice-yearly for oversight and decision-making.

### Ethical Considerations

#### Ethical Approval

On 1 March 2021, the Bauchi State Health Research Ethics Committee approved the overall project (NREC/03/11/19B/2021/03). On 13 September 2021, the Faculty of Medicine and Health Sciences Institutional Review Board at McGill University (A09-B51-21B) approved the initial qualitative phase of focus groups and FCM and will be asked to approve the baseline and follow-up survey and implementation of the intervention of evidence-based dialogues.

#### Consent

Local research supervisors will seek consent from community leaders to work in each community. Facilitators will seek oral informed consent from group participants and parental/guardian consent for participants under 18 years old. Facilitators will ask participants to respect each other’s confidentiality and stress that they should not share personal information in the group setting. For the household survey, interviewers will seek and record on the handset oral informed consent from all respondents. They will also seek parental consent for adolescents under 18 years old.

#### Confidentiality and Data Access

Parents or guardians will not be present during adolescent group activities or individual interviews. We will not record any names or identifying information alongside responses from individuals. Group reports will not identify individuals. Fieldworkers will conduct group sessions in a private location. They will not proceed with household interviews unless they can establish and maintain privacy. Data on the server are password protected; only designated research team members will have access.

#### Minimizing Potential Harms

Survey respondents, especially females, who disclose sensitive issues, like domestic violence, might face retribution if other household members hear of this. Ensuring privacy minimizes this risk. Discussing topics like experiencing violence or abuse might cause distress. Interviewers will carry details of local support services. Facilitators will refer any group participant who is disturbed by the discussion to pre-arranged support in the community.

Fieldworkers, particularly women, potentially face security threats. All fieldworkers will be from the local area. Each fieldwork team will include male members, part of whose role is to ensure the safety of their female colleagues. Government focal points in each ward will advise on any current security risks, and the teams will not visit insecure communities.

### Dissemination and Capacity Building

The Bauchi State Primary Health Care Development Agency will convene meetings at state and zonal levels to discuss findings and policy implications with planners and decision-makers in government, NGOs, and development partners. In year three, the project will share findings from the FCM and baseline survey, and in year five, discuss the impact of the SEPA intervention.

Evidence about factors related to ASRH outcomes in Nigeria and how these might be improved will fill a gap in the literature. We will also publish articles about participatory methods and advances in their analysis, of interest beyond the field of ASRH research. We plan to publish at least five open access papers in peer-reviewed journals and present findings in two international conferences.

#### Capacity Building

The mainly female research team will consolidate their skills in participatory methods and learn about recent advances. The senior team members will support junior members and knowledge users to analyze and present findings and write articles for publication. Officers from the State Ministry of Health and Primary Health Care Development Agency will work on the project and learn about participatory, qualitative methods and collecting reliable survey data. In year five, the research team will facilitate an analysis and interpretation workshop over 18 days for 20 women and men from the government and FOMWAN. Participants will practice analysis techniques using project data. The research team will support them in preparing articles for publication. Adolescents will design materials to share the evidence from FCM and the baseline survey, including video docudramas. We will help them to create videos and other materials, and they will build skills as they do so.

## Results

Focus groups exploring ASRH concerns of stakeholders began in October 2021. Baseline data collection in the household survey is expected to take place in mid-2022.

## Discussion

The project will demonstrate the feasibility of implementing ASRH interventions in a Muslim and conservative culture. It tests a participatory approach: adolescents and other stakeholders share their knowledge, codesign instruments to collect data about ASRH and share the findings, participate in evidence-informed dialogue, and codesign and implement culturally appropriate solutions.

Methodological innovations will have wider relevance. We will test FCM with adolescents and continue our advances in the analysis of these maps. We will refine our use of transitive closure to analyze shifts in intermediate outcomes after an intervention [89].

During the project, adolescents and other stakeholders will identify interventions at different levels, from individual to policy level, to improve ASRH in Bauchi, that can be implemented by adolescents, by communities, and by services. Some will be implemented and have a measurable impact during the project; others are long-term interventions, and measurement of their impact is beyond the scope of this project. The interventions will be specific to the culture of Bauchi, but the way we develop them with stakeholders could have much wider resonance. The project could pave the way for a full-scale randomized controlled trial of the participatory intervention across different contexts.

By training and working with planners and decision-makers, the project will support the adoption of evidence-based policies to improve ASRH in Bauchi. It will contribute to a culture of evidence-based planning of health services, building on previous work in the State by the research team.

Evidence about factors related to ASRH outcomes in Nigeria and implementation and testing of a dialogic intervention to improve these outcomes will fill a gap in the literature. The project will document and test the effectiveness of a participatory approach to ASRH intervention research.

## Appendix

Multimedia Appendix 1 Peer review report from the CIHR/ISRC (Canada) Project Grant Committee.

## Abbreviations

ASRH: adolescent sexual and reproductive health
CASCADA: conscious knowledge, attitude, subjective norms, intention to change, agency to change, discussion of issues, and action to change
FCM: fuzzy cognitive mapping
FOMWAN: Federation of Muslim Women’s Associations in Nigeria
NGO: nongovernment organization
SEPA: socializing evidence for participatory action
