# Supplementary material for: Impact-Oriented Dialogue for Culturally Safe Adolescent Sexual and Reproductive Health in Bauchi State, Nigeria: Protocol for a Codesigned Pragmatic Cluster Randomized Controlled Trial
Source: JMIR Res Protoc. 2022 Mar 15;11(3):e36060. doi: 10.2196/36060 (PMC8965671; doi:10.2196/36060)
Supplement: Multimedia Appendix 1 [file resprot_v11i3e36060_app1.pdf]

**Review Type / Type d'évaluation:**	Reviewer 1 / Évaluateur 1
**Name of Applicant / Nom du chercheur:**	Cockcroft, Anne
**Application No. / Numéro de demande:**	461832
**Agency / Agence:**	CIHR/IRSC
**Competition / Concours:**	Project Grant/Subvention Projet
**Committee / Comité:**	Public, Community & Population Health 2/Santé publique, santé communautaire et santé des populations 2
**Title / Titre:**	Impact oriented dialogue for culturally safe adolescent sexual and reproductive health in Bauchi State, Nigeria

Adjudication Criteria/Critères de sélection

Initial Score/Cote Initiale: 4.4

Top/Bottom Selection/Groupe supérieur/inférieur

- (●) **Top/Groupe supérieur**
- ( ) **Bottom/Groupe inférieur**

**Review Type / Type d'évaluation:**	Reviewer 1 / Évaluateur 1
**Name of Applicant / Nom du chercheur:**	Cockcroft, Anne
**Application No. / Numéro de demande:**	461832
**Agency / Agence:**	CIHR/IRSC
**Competition / Concours:**	Project Grant/Subvention Projet
**Committee / Comité:**	Public, Community & Population Health 2/Santé publique, santé communautaire et santé des populations 2
**Title / Titre:**	Impact oriented dialogue for culturally safe adolescent sexual and reproductive health in Bauchi State, Nigeria

Summary of Application/Résumé de la demande:

The purpose of this project is to co-design culturally appropriate interventions to improve adolescent sexual and reproductive health (ASRH) and pilot their implementation in Bauchi State, Nigeria; a dominant Muslim community. The specific objectives are to explore stakeholder concerns related to ASRH, quantify ASRH related knowledge, attitudes, experiences, and behaviors of adolescents, parents, and service providers engaging this group in dialogue about the evidence and using a participatory approach to develop an intervention. They propose to evaluate the intervention process and impact on ASRH knowledge, attitudes, experiences, and behaviors of male and female adolescents. They further proposed dissemination of the research findings and to build local capacities.

The study used a sequential mixed method design. This is a second submission.

**Review Type / Type d'évaluation:**	Reviewer 1 / Évaluateur 1
**Name of Applicant / Nom du chercheur:**	Cockcroft, Anne
**Application No. / Numéro de demande:**	461832
**Agency / Agence:**	CIHR/IRSC
**Competition / Concours:**	Project Grant/Subvention Projet
**Committee / Comité:**	Public, Community & Population Health 2/Santé publique, santé communautaire et santé des populations 2
**Title / Titre:**	Impact oriented dialogue for culturally safe adolescent sexual and reproductive health in Bauchi State, Nigeria

Strengths and Weaknesses/Forces et faiblesses:

Strengths

The significance of the applicant is well described and the background made a compelling reason why this research is important. One of the most significant strengths of the application is the well-experienced team and their long-term engagement is the province where this proposed is proposed to commence. Another strength is the capacity building that includes various stakeholders that I believe contribute to further research and the sustainability of the intervention. The ethical, risk and mitigations considerations are well described.

Most of the concerns from previous reviewers have been addressed comprehensively.

Weaknesses

The research team proposed the Theory of Planned Behavior CASCADE and although it has been used previously in multiple intervention studies, there is also critique against this theory for example the emphasis on knowledge and behavior. It ignores other intersecting and larger contextual issues for example the influence of culture, poverty, and other SDOH, ethnicity, religions, gender, power. etc. In the previous review, the use of the Theory of Planned Behaviors was also critiqued and it was addressed very superficially in the resubmitted application. They added a sentence related to patriarchy and power differences without really addressing how they will address the structural and contextual issues. That said they partially address it by using the concept ‘choice ability’.

The research team uses the term cultural safety and although it is appropriate, there has recently been critique against the use of this term. As the group continues with this project, I recommend that they look at the different terms; cultural safety and cultural competency versus cultural humility.

During the analysis, they proposed to use Guba’s model to ensure trustworthiness. This model is outdated and critiqued intensively. I proposed to used more recent strategies that include reflexivity, consistency, etc. (see: Morse, M. J. (2015). Critical analysis of strategies for determining rigor in qualitative inquiry. Qualitative Health Research, 25(9), 1212-1222. doi:10.1177/1049732315588501).

I am not sure how the SEPA is defined as an intervention.

I am still unsure about the management of the project. In some places, the team indicated that the research will be conducted virtual but they emphasize that travelling to Bauchi State is safe. I believe some visits to the research environment will be necessary. Virtual supervision only, in such a complex project will not be sufficient.

**Review Type / Type d'évaluation:**	Reviewer 1 / Évaluateur 1
**Name of Applicant / Nom du chercheur:**	Cockcroft, Anne
**Application No. / Numéro de demande:**	461832
**Agency / Agence:**	CIHR/IRSC
**Competition / Concours:**	Project Grant/Subvention Projet
**Committee / Comité:**	Public, Community & Population Health 2/Santé publique, santé communautaire et santé des populations 2
**Title / Titre:**	Impact oriented dialogue for culturally safe adolescent sexual and reproductive health in Bauchi State, Nigeria

Budget Recommendation/Recommandation budgétaire:

I understand that a study of this magnitude is costly. Many items are not well motivated, for example, the payment of a team leader; is that investigator on the grand.

Some payment is not equitable, for example, a post doc receives $70000 per annum but the local research manager only $15000. Both will support managing the project.

Most of the costs are not justified/ itemized.

Please indicate your appraisal of the integration of sex as a biological variable as a strength, weakness, or not applicable to the proposal./Prière de sélectionner une option pour donner votre évaluation de l'intégration du sexe comme variable biologique en tant que point fort ou point faible de la proposition, ou en tant qu'élément non applicable à la proposition.

- (●) **Strength/Point fort**
- ( ) **Weakness/Point faible**
- ( ) **Not applicable/Non applicable**

Please indicate your appraisal of the integration of gender as a socio-cultural determinant of health as a strength, weakness, or not applicable to the proposal./Prière de sélectionner une option pour donner votre évaluation de l'intégration du genre comme déterminant socioculturel de la santé en tant que point fort ou point faible de la proposition, ou en tant qu'élément non applicable à la proposition.

- (●) **Strength/Point fort**
- ( ) **Weakness/Point faible**
- ( ) **Not applicable/Non applicable**

**Review Type / Type d'évaluation:**	Reviewer 1 / Évaluateur 1
**Name of Applicant / Nom du chercheur:**	Cockcroft, Anne
**Application No. / Numéro de demande:**	461832
**Agency / Agence:**	CIHR/IRSC
**Competition / Concours:**	Project Grant/Subvention Projet
**Committee / Comité:**	Public, Community & Population Health 2/Santé publique, santé communautaire et santé des populations 2
**Title / Titre:**	Impact oriented dialogue for culturally safe adolescent sexual and reproductive health in Bauchi State, Nigeria

Sex and/or Gender Considerations/Notions de sexe et/ou de genre:

Gender and biological sex difference are addressed sufficiently. The team will report on the difference between the fuzzy cognitive maps created by boys and girls. Both male and female adolescents will be part of all phases of the study. Strategies are developed to address sensitive issues and mitigate safety concerns related to discussion between males and females related to SRH of adolescents.

**Review Type / Type d'évaluation:**	Reviewer 2 / Évaluateur 2
**Name of Applicant / Nom du chercheur:**	Cockcroft, Anne
**Application No. / Numéro de demande:**	461832
**Agency / Agence:**	CIHR/IRSC
**Competition / Concours:**	Project Grant/Subvention Projet
**Committee / Comité:**	Public, Community & Population Health 2/Santé publique, santé communautaire et santé des populations 2
**Title / Titre:**	Impact oriented dialogue for culturally safe adolescent sexual and reproductive health in Bauchi State, Nigeria

Adjudication Criteria/Critères de sélection

Initial Score/Cote Initiale: 4.2

Top/Bottom Selection/Groupe supérieur/inférieur

- (●) **Top/Groupe supérieur**
- ( ) **Bottom/Groupe inférieur**

**Review Type / Type d'évaluation:**	Reviewer 2 / Évaluateur 2
**Name of Applicant / Nom du chercheur:**	Cockcroft, Anne
**Application No. / Numéro de demande:**	461832
**Agency / Agence:**	CIHR/IRSC
**Competition / Concours:**	Project Grant/Subvention Projet
**Committee / Comité:**	Public, Community & Population Health 2/Santé publique, santé communautaire et santé des populations 2
**Title / Titre:**	Impact oriented dialogue for culturally safe adolescent sexual and reproductive health in Bauchi State, Nigeria

Summary of Application/Résumé de la demande:

This is a participatory research project using a sequential mixed methods design. The objectives are to:

(1)Explore priority stakeholder concerns about ASRH in Bauchi, collate the knowledge of female and male adolescents and other stakeholders about causes and protective factors for these concerns, and compare their knowledge with documented associations in the literature;
(2)Quantify ASRH-related knowledge, attitudes, experiences and behaviours of female and male adolescents, parents, and service providers, using data collection instruments informed by the collated local knowledge and literature review;
(3)Engage adolescents, parents, service providers and decision makers in dialogue about the evidence on ASRH outcomes and causes, to identify and implement locally appropriate interventions at different levels to improve ASRH;
(4)Evaluate the intervention process and measure impact on ASRH knowledge, attitudes, experiences and behaviours of female and male adolescents and other stakeholders quantitatively and qualitatively;
(5)Disseminate the research findings more widely focusing on (i) participatory methods for contextualising ASRH interventions and (ii) the impact of the interventions in Bauchi; and
(6)Build local capacities to gather stakeholder evidence about health-related concerns, implement locally relevant interventions, and measure their impact.

Strengths and Weaknesses/Forces et faiblesses:

Clinical significance:

The applicants provide a strong rationale for the proposal, citing the high rates of maternal mortality in Nigeria, and the fact that adolescents represent a large proportion of the population and have high pregnancy rates.

Methodological strengths:

Participatory approach: A strength of the study is its participatory approach. Adolescents, parents, religious leaders, and providers will be involved in every step of the study, from identification of risk factors, to sharing of information, and development of an intervention to address ASRH concerns.

Capacity building: Government officers will also be trained in data analysis and interpretation, which will contribute to a culture of evidence-based planning of health services.

Partnerships: Another significant strength is its existing partnerships with relevant knowledge users, including the Federation of Muslim Women's Association in Nigeria and the Bauchi State Primary Health Care Development Agency, which have been partners since 2009. They previously partnered with these two groups on a culturally safe study on child spacing, opening the door to this study on ASRH. They also have buy-in from the Muslim community, including the Chief Imam of Bauchi.

Theoretical framework: The applicants use the "CASCADA results chain" to understand the relationships between knowledge, attitudes, and practice. The applicants have used in resource-poor settings previously. The applicants also incorporate ideas of cultural safety in their framework.

Pilot funding: The applicants have pilot funding from the CIHR IPPH Priority Announcement for Global Health, which will fund focus groups to identify priority concerns for ASRH. This, along with a systematic review will be the basis for the first phase of the research, which is "fuzzy cognitive mapping" to depict factors that stakeholders consider to be causes of an outcome.

Methodological weaknesses:

Systematic review: Objective 1 is listed as being a systematic review to inform the fuzzy cognitive mapping. This section did not provide sufficient detail to evaluate the rigour of the approach, nor what this systematic review would add to the literature, given that the applicants cite a previous review with over 1,000 articles on the same topic.

Lack of detail on some of the methods: This is an extremely ambitious proposal, which leaves little room to describe in detail some of the methods. For example, under fuzzy cognitive mapping: how does transitive closure analysis work and

why is it appropriate here? How will conflicting priorities identified by different groups be handled? Under baseline survey, what specific items will parents complete vs. adolescents, and how do the surveys differ for these groups? What role do the key informant interviews play and how do these differ from the fuzzy cognitive mapping work? Under Socializing evidence for participatory action, what will the intervention be like? I realize much of this will be determined by the results of the other parts of the study, but some examples of what it could look like would be helpful for evaluating its feasibility.

Participant recruitment: From what I can tell, all aspects of the research will take place in a fairly contained region of Nigeria (60 communities across 6 wards). There is ambitious recruitment at each phase of the study (e.g., fuzzy cognitive mapping, survey, integrated KT activities, intervention development and evaluation). Will individuals who participated in one aspect of the study be eligible for another part of the study? If so, what impact does that have on the results?

Response bias: How will response bias influence the results and their interpretation? Is it possible that, especially in evaluation of the intervention, that certain “negative” outcomes will be under-reported, particularly in the communities that received the intervention?

Knowledge translation:

Knowledge translation is built into the research design, with buy-in from important stakeholders (adolescents, parents, community leaders, service providers, etc) at every stage, increasing the potential impact and utility of results.

Study team:

The study team includes senior investigators, trainees, and several knowledge users. The NPA has 20+ years of experience conducting research related to ASRH in similar regions, with specific experience working in the proposed communities in Nigeria. There is a knowledge user PA who will be instrumental in leading the work in Nigeria.

**Review Type / Type d'évaluation:**	Reviewer 2 / Évaluateur 2
**Name of Applicant / Nom du chercheur:**	Cockcroft, Anne
**Application No. / Numéro de demande:**	461832
**Agency / Agence:**	CIHR/IRSC
**Competition / Concours:**	Project Grant/Subvention Projet
**Committee / Comité:**	Public, Community & Population Health 2/Santé publique, santé communautaire et santé des populations 2
**Title / Titre:**	Impact oriented dialogue for culturally safe adolescent sexual and reproductive health in Bauchi State, Nigeria

Budget Recommendation/Recommandation budgétaire:

The budget is comprehensive and appears appropriate.

Please indicate your appraisal of the integration of sex as a biological variable as a strength, weakness, or not applicable to the proposal./Prière de sélectionner une option pour donner votre évaluation de l'intégration du sexe comme variable biologique en tant que point fort ou point faible de la proposition, ou en tant qu'élément non applicable à la proposition.

- ○ **Strength/Point fort**
- ○ **Weakness/Point faible**
- ◉ **Not applicable/Non applicable**

Please indicate your appraisal of the integration of gender as a socio-cultural determinant of health as a strength, weakness, or not applicable to the proposal./Prière de sélectionner une option pour donner votre évaluation de l'intégration du genre comme déterminant socioculturel de la santé en tant que point fort ou point faible de la proposition, ou en tant qu'élément non applicable à la proposition.

- ◉ **Strength/Point fort**
- ○ **Weakness/Point faible**
- ○ **Not applicable/Non applicable**

Sex and/or Gender Considerations/Notions de sexe et/ou de genre:

The proposal will address gender, but not sex. It amplifies voices of adolescent girls in collecting and sharing information about risks to their sexual and reproductive health. The survey sampling method will ensure that there is at least one girl per household included, and the analysis will "include gender as a key variable in examination of ASRH outcomes" (however, I would have liked to see how this will be done – e.g., by adjustment or stratification).

**Review Type / Type d'évaluation:**	Reviewer 3 / Évaluateur 3
**Name of Applicant / Nom du chercheur:**	Cockcroft, Anne
**Application No. / Numéro de demande:**	461832
**Agency / Agence:**	CIHR/IRSC
**Competition / Concours:**	Project Grant/Subvention Projet
**Committee / Comité:**	Public, Community & Population Health 2/Santé publique, santé communautaire et santé des populations 2
**Title / Titre:**	Impact oriented dialogue for culturally safe adolescent sexual and reproductive health in Bauchi State, Nigeria

Adjudication Criteria/Critères de sélection

Initial Score/Cote Initiale: 4.1

Top/Bottom Selection/Groupe supérieur/inférieur

- (●) **Top/Groupe supérieur**
- ( ) **Bottom/Groupe inférieur**

**Review Type / Type d'évaluation:**	Reviewer 3 / Évaluateur 3
**Name of Applicant / Nom du chercheur:**	Cockcroft, Anne
**Application No. / Numéro de demande:**	461832
**Agency / Agence:**	CIHR/IRSC
**Competition / Concours:**	Project Grant/Subvention Projet
**Committee / Comité:**	Public, Community & Population Health 2/Santé publique, santé communautaire et santé des populations 2
**Title / Titre:**	Impact oriented dialogue for culturally safe adolescent sexual and reproductive health in Bauchi State, Nigeria

Summary of Application/Résumé de la demande:

Adolescent sexual health and reproductive health (ASHR) is a key public health priority in Nigeria, where rates of early childbearing, unsafe abortions, HIV risk, and sexual violence are high. This project will use participatory research to collect qualitative and quantitative data on ASRH among married and unmarried adolescent girls and boys (aged 10-19) and co-design with adolescents and other stakeholders interventions to address ASRH. The team's research builds on a strong background in conducting participatory research in the Bauchi State of Nigeria, where the proposed research will be conducted.

Objectives

1. Explore priority stakeholder concerns about ASRH in Bauchi, collate the knowledge of female and male adolescents and other stakeholders about causes and protective factors for these concerns, and compare their knowledge with documented associations in the literature.
2. Quantify ASRH-related knowledge, attitudes, experiences and behaviours of female and male adolescents, parents, and service providers, using data collection instruments informed by the collated local knowledge and literature review.
3. Engage adolescents, parents, service providers and decision makers in dialogue about the evidence on ASRH outcomes and causes, to identify and implement locally appropriate interventions at different levels to improve ASRH.
4. Evaluate the intervention process and measure impact on ASRH knowledge, attitudes, experiences and behaviours of female and male adolescents and other stakeholders quantitatively and qualitatively.
5. Disseminate the research findings more widely focusing on (i) participatory methods for contextualising ASRH interventions and (ii) the impact of the interventions in Bauchi.
6. Build local capacities to gather stakeholder evidence about health-related concerns, implement locally relevant interventions, and measure their impact. Train a doctoral student at McGill.

The team draws on the CASCADA model (Conscious knowledge, Attitudes, Subjective norms, intention to Change, Agency Discussion and Action), which is based on the Theory of Planned Behaviour, to inform the design of the intervention. They also draw on a concept of choice disability among marginalized women, pointing to the need for for interventions focusing on empowerment and enabling supports to promote individual choices.

Methods

The team will use a sequential mixed methods design, including qualitative and quantitative data collection phase as well as participatory action to co-design interventions. The project also includes a final evaluation using quantitative and qualitative methods.

Objective 1: Focus groups (to be completed with other funding), fuzzy cognitive mapping (FCM; graphic representation of knowledge about causality in a system) to map factors believed to cause the priority ASRH concerns identified in the focus groups. FCM will be conducted with adolescent groups (separately by age and gender), adults, traditional and religious leaders, and health service providers in 6 communities, including urban, rural, and rural-remote.

Objective 2: Baseline household survey of parents and adolescents (6,000 adolescent girls and 4,000 adolescent boys, 4,000 mothers and 2,500 fathers) in 60 communities in 6 wards which will focus on “knowledge, attitudes, and experience amenable to change”, informed by the focus groups

Objective 3: Three of 6 wards (in 30 communities) will be randomly selected to receive Socializing evidence for participatory action (SEPA), which involves dialogues with stakeholders to review evidence and develop local interventions. Docudramas developed with youth will be used to share evidence on maps from objective 1. SEPA communities (adolescent girls, boys, adults, community and religious leaders, and relevant service providers) will develop local actions based on evidence. Special steps will be taken to ensure that the perspectives of females are listened to and respected.

Objective 4:
Process evaluation – towards end of implementation, participants will review SEPA activities and implementation will be scored. Outcome evaluation – follow-up survey in the same communities. Qualitative evaluation - Most Significant Change technique (MSC), collect stories from participants of life changes attributed to SEPA, involving about 60 story (25+ adolescent girls and 15+ boys).

Findings will be disseminated in Bauchi communities through meetings with the Bauchi State Primary Health Care Development Agency (BaSPHCDA).

Capacity building: The team will hold analysis and interpretation workshops and adolescents will design materials to share the evidence.

**Review Type / Type d'évaluation:**	Reviewer 3 / Évaluateur 3
**Name of Applicant / Nom du chercheur:**	Cockcroft, Anne
**Application No. / Numéro de demande:**	461832
**Agency / Agence:**	CIHR/IRSC
**Competition / Concours:**	Project Grant/Subvention Projet
**Committee / Comité:**	Public, Community & Population Health 2/Santé publique, santé communautaire et santé des populations 2
**Title / Titre:**	Impact oriented dialogue for culturally safe adolescent sexual and reproductive health in Bauchi State, Nigeria

Strengths and Weaknesses/Forces et faiblesses:

The team is strong. The NPA has extensive experience conducting community-based participatory research and has worked closely with stakeholders in Nigeria and the state of Bauchi in particular. The team includes Co-PA knowledge user who is the former Director of Nursing Services and Amirah of FOMWAN in Bauchi, and is now heading its Health Committee.

The scope of the project on sexual and reproductive health is, perhaps necessarily, very broad as it touches on HIV risk, unwanted pregnancy, abortion, and sexual violence. Each of these separate outcomes is complex and comes with a large literature. For example, a focus on gender-based violence requires extensive knowledge regarding its causes and best practices for prevention. As such, this is an extremely complex and ambitious endeavour.

If the ultimate goal is to improve the sexual health of girls, it would seem important to consider the literature on effective interventions that have been used in low income countries and draw on this knowledge in the development of interventions and perhaps shared with community stakeholders to inform their planning.

Importantly, while the introduction draws attention to the sexual health of girls, the research plan focuses on both boys and girls (although a larger attention is given to girls), who will participate in all phases of the project. Again, this makes the project very ambitious, challenging and less focused.

Given concerns from previous reviewers that the voices of adolescents may be overshadowed by those of other stakeholders, the team notes that the perspectives of adolescent girls & boys will be given equal weight with those of stakeholders. The perspectives of adolescents should actually be prioritized rather than given equal weight with that of other stakeholders. Ultimately, if the interventions are not meaningful to the target population (i.e., youth) they will not be effective – therefore, engagement with youth is of most importance.

The evaluation component will simply test the overall impact of SEPA (the engagement process), with data pooled across communities, rather than test the effectiveness of the interventions themselves. With SEPA, if every community in the SEPA arm develops its own action plan the interventions are likely to be different in every community. Is there a way to determine which intervention is most effective? Can you take the best of all interventions for scale up across the region? Or will all interventions remain locally specific? Are there learnings from each local area that could be shared in developing an overarching prevention plan? Obviously, the communities in the control arm of the project will not receive SEPA. Are there plans to implement SEPA in the control arm? Perhaps a step-wedge design would be more appropriate.

Additionally, project may benefit from a clearer focus specifically on the sexual health of girls and women and providing a theoretical framing and research around this topic rather than more generally around boys and girls. It may also make sense to engage adult women who have experienced sexual health problems and survivors of gender based violence in the development of interventions. Involving parents in intervention planning may be problematic if they are the perpetrators.

**Review Type / Type d'évaluation:**	Reviewer 3 / Évaluateur 3
**Name of Applicant / Nom du chercheur:**	Cockcroft, Anne
**Application No. / Numéro de demande:**	461832
**Agency / Agence:**	CIHR/IRSC
**Competition / Concours:**	Project Grant/Subvention Projet
**Committee / Comité:**	Public, Community & Population Health 2/Santé publique, santé communautaire et santé des populations 2
**Title / Titre:**	Impact oriented dialogue for culturally safe adolescent sexual and reproductive health in Bauchi State, Nigeria

As mentioned in the sex/gender section, some improvement is needed in integrating sex and gender into the research.

**Review Type / Type d'évaluation:**	Reviewer 3 / Évaluateur 3
**Name of Applicant / Nom du chercheur:**	Cockcroft, Anne
**Application No. / Numéro de demande:**	461832
**Agency / Agence:**	CIHR/IRSC
**Competition / Concours:**	Project Grant/Subvention Projet
**Committee / Comité:**	Public, Community & Population Health 2/Santé publique, santé communautaire et santé des populations 2
**Title / Titre:**	Impact oriented dialogue for culturally safe adolescent sexual and reproductive health in Bauchi State, Nigeria

Budget Recommendation/Recommandation budgétaire:

Large budget but appropriate given scope and multi-phased data collection. Surprised that only one post-doc and no other trainees at McGill are proposed.

Please indicate your appraisal of the integration of sex as a biological variable as a strength, weakness, or not applicable to the proposal./Prière de sélectionner une option pour donner votre évaluation de l'intégration du sexe comme variable biologique en tant que point fort ou point faible de la proposition, ou en tant qu'élément non applicable à la proposition.

○ **Strength/Point fort**
◉ **Weakness/Point faible**
○ **Not applicable/Non applicable**

Please indicate your appraisal of the integration of gender as a socio-cultural determinant of health as a strength, weakness, or not applicable to the proposal./Prière de sélectionner une option pour donner votre évaluation de l'intégration du genre comme déterminant socioculturel de la santé en tant que point fort ou point faible de la proposition, ou en tant qu'élément non applicable à la proposition.

○ **Strength/Point fort**
◉ **Weakness/Point faible**
○ **Not applicable/Non applicable**

**Review Type / Type d'évaluation:**	Reviewer 3 / Évaluateur 3
**Name of Applicant / Nom du chercheur:**	Cockcroft, Anne
**Application No. / Numéro de demande:**	461832
**Agency / Agence:**	CIHR/IRSC
**Competition / Concours:**	Project Grant/Subvention Projet
**Committee / Comité:**	Public, Community & Population Health 2/Santé publique, santé communautaire et santé des populations 2
**Title / Titre:**	Impact oriented dialogue for culturally safe adolescent sexual and reproductive health in Bauchi State, Nigeria

Sex and/or Gender Considerations/Notions de sexe et/ou de genre:

The team makes a strong attempt to integrate gender into the proposal. However, to fully consider sex (which should also be considered) and gender into this work, one would need to bring in theory (beyond mentioning choice disability) that focuses on gender roles, gender norms and patriarchal structures that affect sexual and reproductive health. For example, for male adolescents, what theory and evidence drives our understanding of their sexual health, such as for example, their likelihood of engaging in unprotected sex and gender-based violence? This background is important for framing the development of effective interventions.

Review Type/Type d'évaluation: SO Notes /Notes de l'agent scientifique
Name of Applicant/Nom du chercheur: Cockcroft, Anne
Application No./Numéro de demande: 461832
Agency/Agence: CIHR/IRSC
Competition/Concours: 2021-04-01 Project Grant/Subvention Projet
Committee/Comité: Public, Community & Population Health 2/Santé publique, santé communautaire et santé des populations 2
Title/Titre: Impact oriented dialogue for culturally safe adolescent sexual and reproductive health in Bauchi State, Nigeria

---

Assessment/Évaluation:

Strengths (including SGBA considerations):

· Well described proposal with strong rationale.

· Experienced team and meaningful collaborations in place.

· Community participatory approach was strong, and the team has established connections.

· Overall the team responded to previous concerns.

Weaknesses (including SGBA considerations):

· Social determinants of health are not explicitly considered in the theoretical framework and could be further developed in the proposal.

· There are many objectives and methods details that were lacking in places.

· Scope is large with many outcomes; it may be a challenge to study all the complex outcomes proposed adequately.

· It remains unclear how some of the power dynamics and patriarchy will be handled.

· The proposal addresses the evaluation of the engagement process versus the interventions themselves; however, this project does not specifically speak to the effectiveness of the interventions themselves.

· Consider the equity of the payment between the local and non-local staff doing the same role (e.g. postdoc versus local research coordinator)

· The budget justification was lacking in places.

Review Type/Type d'évaluation: SO Notes /Notes de l'agent scientifique
Name of Applicant/Nom du chercheur: Cockcroft, Anne
Application No./Numéro de demande: 461832
Agency/Agence: CIHR/IRSC
Competition/Concours: 2021-04-01 Project Grant/Subvention Projet
Committee/Comité: Public, Community & Population Health 2/Santé publique, santé communautaire et santé des populations 2
Title/Titre: Impact oriented dialogue for culturally safe adolescent sexual and reproductive health in Bauchi State, Nigeria

Assessment/Évaluation:

Budget:

- No specific cuts were proposed.

Please see the reviewer notes for additional details.

****
Note: The final rating of the application, provided in the Notice of Recommendation (NOR) and Notice of Decision (NOD), is the averaged rating of the peer review committee members following the discussion of the application during the committee meeting, and therefore may differ from the ratings provided by the assigned reviewers in their respective reviews.

Remarque : La cote définitive de la demande, qui apparaît dans l'avis de recommandation et l'avis de décision, représente la moyenne des cotes accordées par les membres du comité d'évaluation par les pairs après avoir débattu de la demande à la réunion du comité. Elle peut donc différer de celle donnée par les évaluateurs dans leur évaluation respective.

``````````````````````````````````````````````````````
``````````````````````````````````````````````````````
